# Cesarean Section in Patients with Myomas Located on the Anterior Wall of the Cervix

**DOI:** 10.1055/s-0040-1708061

**Published:** 2020-06-05

**Authors:** Yuji Hiramatsu

**Affiliations:** 1 Department of Obstetrics and Gynecology, Okayama City General Medical Center, Kita-Ku, Okayama, Japan

**Keywords:** cesarean section, myoma, myomectomy, incision

## Abstract

In patients with uterine myomas undergoing cesarean section, the site of the uterine incision differs depending on the location, size, and number of myomas and the location of the placenta. The difficulty is particularly high when the myomas are located on the anterior wall of the cervix. In all patients, safe fetal birth is the top priority. The incision is usually made to avoid uterine myomas, and enucleation of the myomas is performed after fetal birth. However, in some patients, the fetus must be delivered after enucleating the myomas, so practice is necessary to be prepared for such cases. In this report, I explain the critical points to be noted at the time of cesarean section, especially in patients with myomas located on the anterior wall of the cervix.

## Strategies

It is important to be aware of the events shown in Table 1 that occur during a cesarean section with myomas. 1 2 3 4 5 6 It should be recognized that multiple myomas or large subserosal myomas can cause the uterus to twist; therefore, the surgical visual field at the time of laparotomy may differ greatly from usual.

**Table 1:** Precautions when performing cesarean section in patient with uterine myomas

1	Abnormal fetal lie or fetal position is likely to occur.
2	Abnormalities in the placental attachment site are likely to occur.
3	Multiple myomas or large subserosal myomas can cause the uterus to twist.
4	If the myoma is located in the anterior or lateral wall of the lower uterus, a normal lower uterine transverse incision may not deliver the baby.
5	The fetal lie may change simultaneously with rupture of the placental membranes.
6	It is important to hold the lowest part or the fetal feet securely after the membranes have ruptured because the lowest part of the fetus is not fixed due to myoma.
7	Thick uterine wall increases blood loss during incision, and it is difficult to expand incision.
8	Myoma may worsen uterine contraction and increase bleeding.

Abnormalities in the placental attachment site are likely to occur secondary to uterine myomas, which often accompany an abnormal fetal lie or position. For this reason, the lowest part is not fixed, and the fetal lie may change simultaneously with rupture of the placental membranes. It is important to hold the lowest part or the fetal feet securely after the membranes have ruptured. In patients with uterine myomas, the uterine muscular layer is thick and extends with difficulty; therefore, it is necessary to widen the incision slightly to minimize pressure on the fetal head. In particular, when delivering in breech presentation, the Veit–Smellie maneuver is recommended to protect the fetus. 4

After fetal delivery, especially when the placenta is attached to the back of the myoma, oxytocin should be administered to reduce the amount of bleeding. 1

Before each operation, it is important to examine which of the potential difficulties shown in Table 1 are likely to occur and to prepare for possible second and third actions before starting surgery. It is important for the operating surgeon to perform preoperative ultrasonography and to consider the fetal lie, placental position, and location, size, and number of myomas. It is also useful to determine the incision site by examining sagittal, horizontal, and coronal magnetic resonance images (MRI) simultaneously. 1 2 3 4

## Preoperative Inspection and Preparation

Blood and urine tests, electrocardiograms, chest X-ray tests, and other examinations can be performed as for myomectomy for nonpregnancy. Preoperative ultrasonography is performed by the operating surgeon, and the incision site and incision method are determined by considering the size, location, and number of myomas, as well as the placental location. The same information is confirmed by MRI, and if there is a myoma in the anterior cervical wall, it is important to check how much the bladder is elevated. In addition, check which part has high blood flow using Doppler ultrasonography. 1

In our department, all myomas are enucleated at the time of cesarean section, so autologous blood is stored for patients with a large myoma or multiple myomas. 6

## Informed Consent

The main points of informed consent are: (1) difficulty in delivering the fetus because of the presence of the uterine myomas, and (2) the possibilities of increased blood loss and complications ( Table 1 ).

## Selecting the Incision Site

**Fig. 1:**
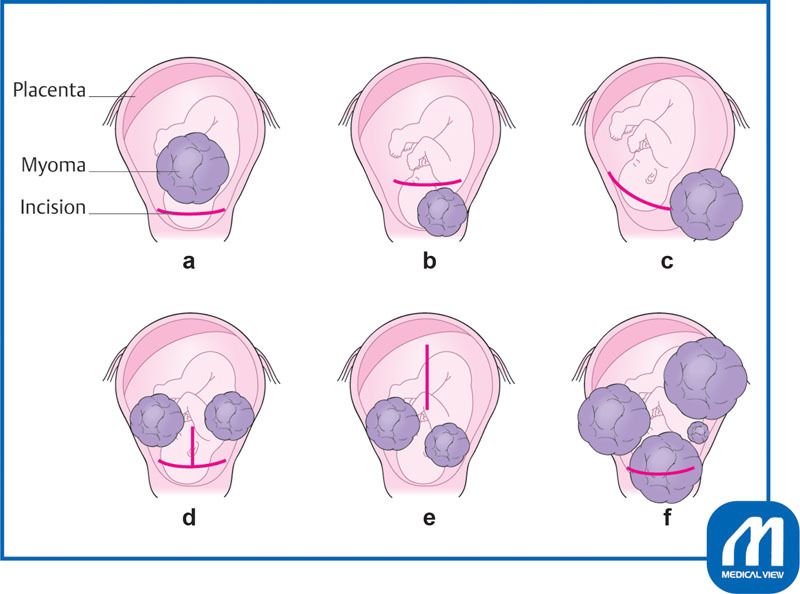
**(a)** Lower transverse incision. **(b)** Lower transverse incision at a slightly higher position. **(c)** Lower *J* -shaped incision. **(d)** Reverse *T* -shaped incision. **(e)** Longitudinal incision. **(f)** Myomectomy and lower transverse incision. (Reproduced with permission of Hiramatsu Y. Cesarean section in patients with myomas located on the anterior wall of the cervix. In: Hiramatsu Y, Konishi I, Sakuragi N, Takeda S, eds. OGS Now, No.11 Uterine Myoma—How Do You Operate in Such a Case? (Japanese). Tokyo: Medical View; 2012:118–125. Copyright © Medical View).

Determining how to deliver the fetus safely is a problem when myomas are located in the lower anterior wall of the uterus. It is necessary to determine the incision site based on preoperative evaluation using ultrasonography and MRI. We check the size, location, and number of myomas as well as the placental location ( Fig. 1 ). The incision options are as follows:

1. Lower transverse incision: the same incision as for a lower segment transverse section ( Fig. 1a ).
2. Lower transverse incision at a slightly higher position: the transverse incision is made at a slightly higher position ( Fig. 1b ).
3. Lower *J* -shaped incision: the incision is made in a *J* -shape to avoid the myomas ( Fig. 1c ).
4. Reverse *T* -shaped incision: a reverse *T* -shaped incision is performed when the fetus cannot be delivered using only the lower transverse incision ( Fig. 1d ).
5. Longitudinal incision: this incision is performed when the fetus cannot be delivered by incisions 1 to 3 described above ( Fig. 1e ).
6. Myomectomy and lower transverse incision: First, enucleate the myoma and then make a lower transverse incision at the bottom of the myoma. This incision does not require enlarging the abdominal wall incision, but is an option only for surgeons who are well experienced in myomectomy during cesarean section or during pregnancy ( Fig. 1f ).

Each of these incisions can be considered, but if the planned incision is not satisfactory, it is necessary to use the preplanned second and third option and to proceed with the operation. With no plan in place after the membranes rupture, the fetal condition will worsen.

## Surgical Technique

In this section, I present three cases and explain the points to be noted in each procedure.

### Case 1

**Fig. 2:**
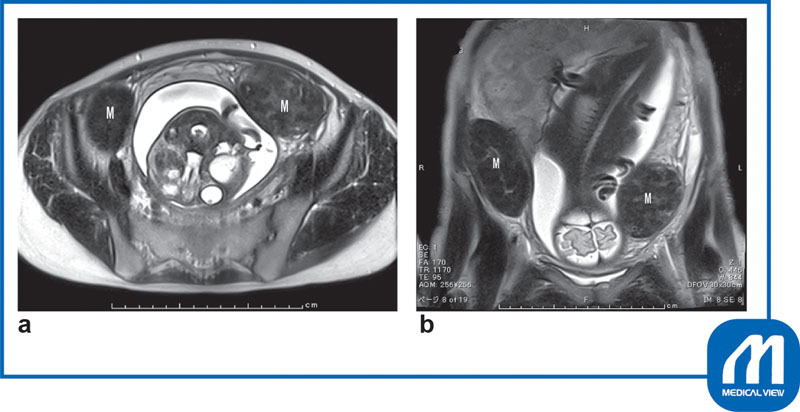
Case 1: magnetic resonance imaging findings (T2W). Two myomas (M) (right 9 × 7 × 5 cm, left 11 × 7 × 6 cm) were found slightly above the uterine isthmus. **(a)** Horizontal image; **(b)** coronal image. (Reproduced with permission of Hiramatsu Y. Cesarean section in patients with myomas located on the anterior wall of the cervix. In: Hiramatsu Y, Konishi I, Sakuragi N, Takeda S, eds. OGS Now, No.11 Uterine Myoma—How Do You Operate in Such a Case? (Japanese). Tokyo: Medical View; 2012:118–125. Copyright © Medical View).

**Fig. 3:**
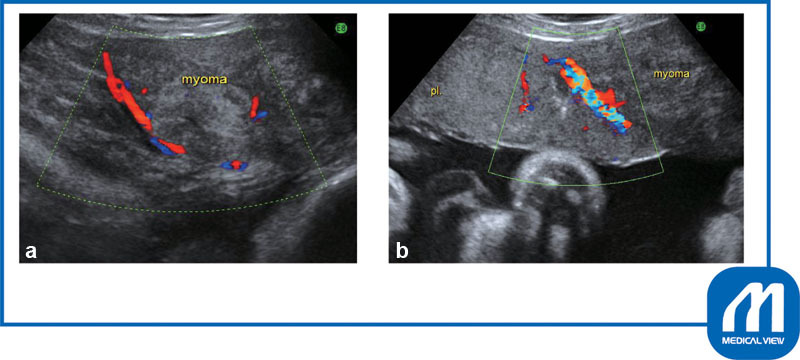
Case 1: Ultrasonography findings. **(a)** right myoma: abundant blood flow was seen around the myoma. **(b)** Left myoma: the placenta was present directly under the right myoma (pl: placenta). (Reproduced with permission of Hiramatsu Y. Cesarean section in patients with myomas located on the anterior wall of the cervix. In: Hiramatsu Y, Konishi I, Sakuragi N, Takeda S, eds. OGS Now, No.11 Uterine Myoma—How Do You Operate in Such a Case? (Japanese). Tokyo: Medical View; 2012:118–125. Copyright © Medical View).

Two myomas (right wall: 9 cm, left wall: 11 cm in size) were found slightly above the uterine isthmus ( Fig. 2 ), and the placenta was present directly under the right myoma ( Fig. 3 ).

*Preoperative judgment* : The fetal head was below the two myomas, so I attempted to deliver the fetus with a normal lower transverse incision ( Fig. 1a ) and planned a reverse T incision if delivery was difficult.

**Fig. 4:**
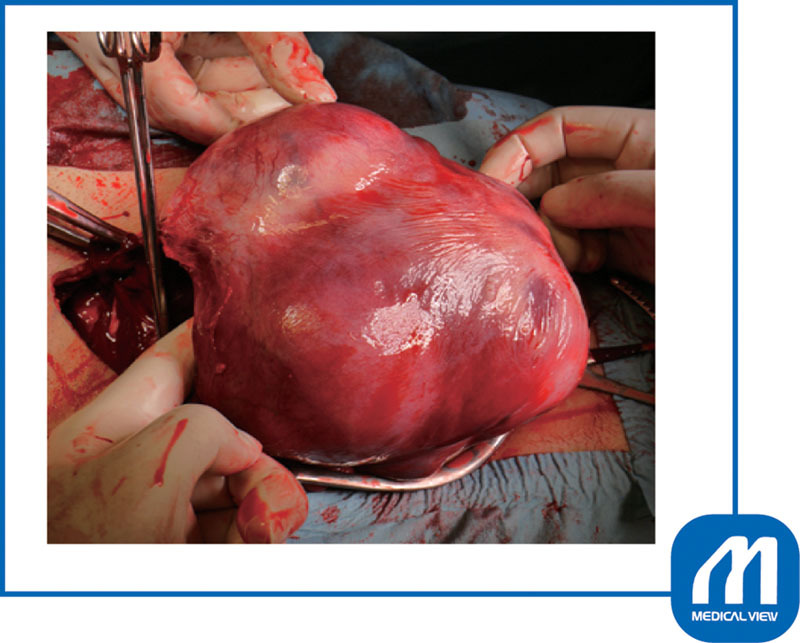
Case 1: condition after the delivery of fetus. Two myomas were in contact with the incision wound. (Reproduced with permission of Hiramatsu Y. Cesarean section in patients with myomas located on the anterior wall of the cervix. In: Hiramatsu Y, Konishi I, Sakuragi N, Takeda S, eds. OGS Now, No.11 Uterine Myoma—How Do You Operate in Such a Case? (Japanese). Tokyo: Medical View; 2012:118–125. Copyright © Medical View).

**Fig. 5:**
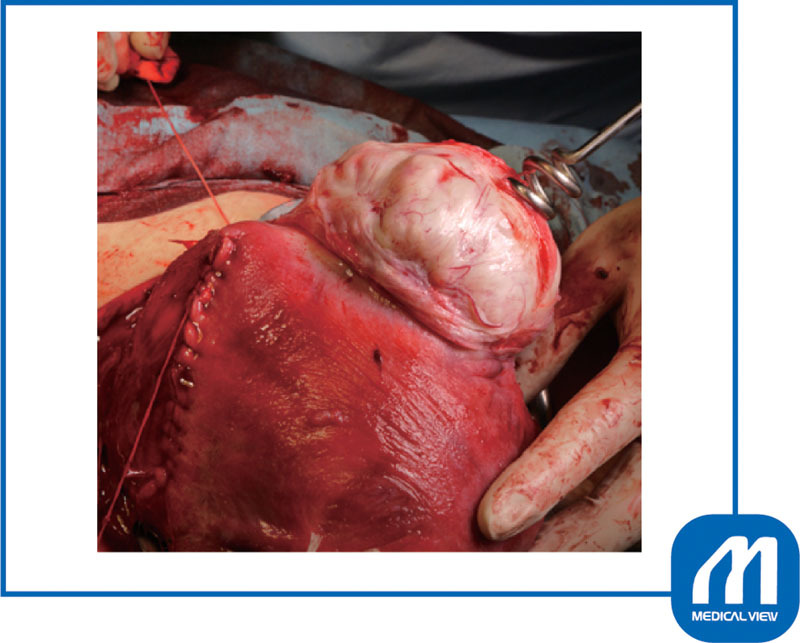
Case 1: myomectomy. The myoma attached under the placenta was enucleated first. If myoma is enucleated in the correct layer, there is little bleeding even during the cesarean section. (Reproduced with permission of Hiramatsu Y. Cesarean section in patients with myomas located on the anterior wall of the cervix. In: Hiramatsu Y, Konishi I, Sakuragi N, Takeda S, eds. OGS Now, No.11 Uterine Myoma—How Do You Operate in Such a Case? (Japanese). Tokyo: Medical View; 2012:118–125. Copyright © Medical View).

**Fig. 6:**
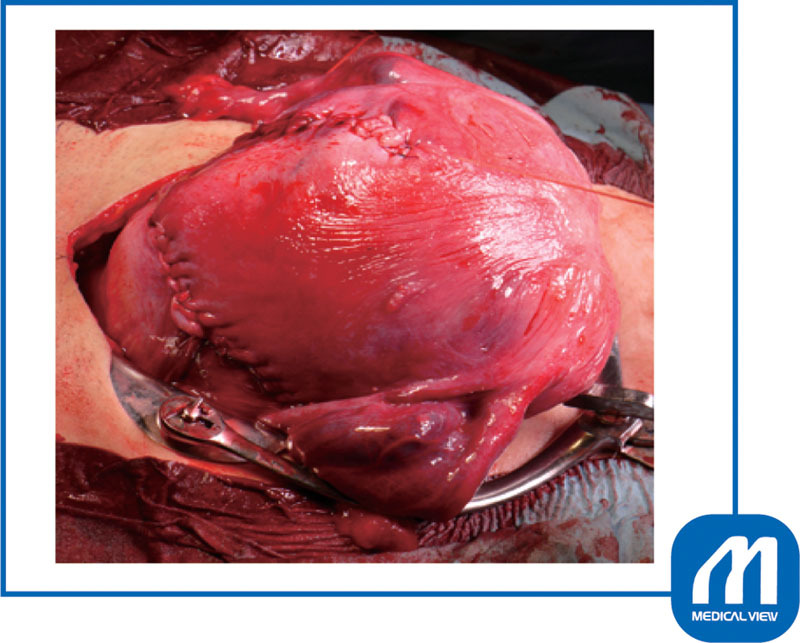
Case 1: condition after myomectomy. The total operation time was 80 minutes, bleeding during cesarean section was 900 mL (including amniotic fluid), and bleeding at the time of myomectomy was 190 mL. (Reproduced with permission of Hiramatsu Y. Cesarean section in patients with myomas located on the anterior wall of the cervix. In: Hiramatsu Y, Konishi I, Sakuragi N, Takeda S, eds. OGS Now, No.11 Uterine Myoma—How Do You Operate in Such a Case? (Japanese). Tokyo: Medical View; 2012:118–125. Copyright © Medical View).

**Fig. 7:**
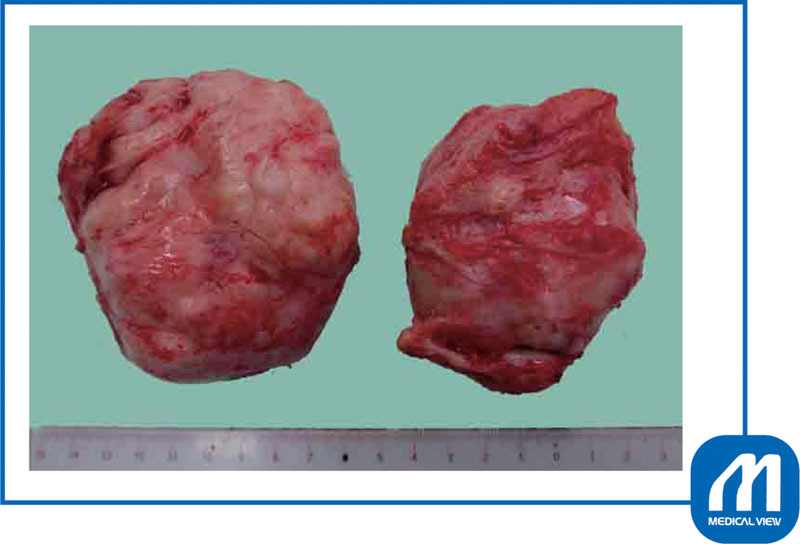
Case 1: enucleated myomas. (Reproduced with permission of Hiramatsu Y. Cesarean section in patients with myomas located on the anterior wall of the cervix. In: Hiramatsu Y, Konishi I, Sakuragi N, Takeda S, eds. OGS Now, No.11 Uterine Myoma—How Do You Operate in Such a Case? (Japanese). Tokyo: Medical View; 2012:118–125. Copyright © Medical View).

*Surgical technique* : After dissecting the bladder, I made a low transverse incision and delivered the fetus without difficulty. Preoperative color Doppler examination showed that the placenta was attached to the back of the myoma and that blood flow to the myoma was abundant ( Fig. 3a ). Therefore, an oxytocin infusion was started at the same time as the umbilical cord clamping, and the placenta was slowly delivered while massaging the uterine fundus. Because the myomas on both sides were in contact with the incision wound ( Fig. 4 ), I sutured both edges of the incision. The myoma attached under the placenta was enucleated first followed by enucleating the other myoma ( Fig. 5 ) and suturing the incision ( Fig. 6 ). Fig. 7 shows that both myomas were enucleated.

### Case 2

**Fig. 8:**
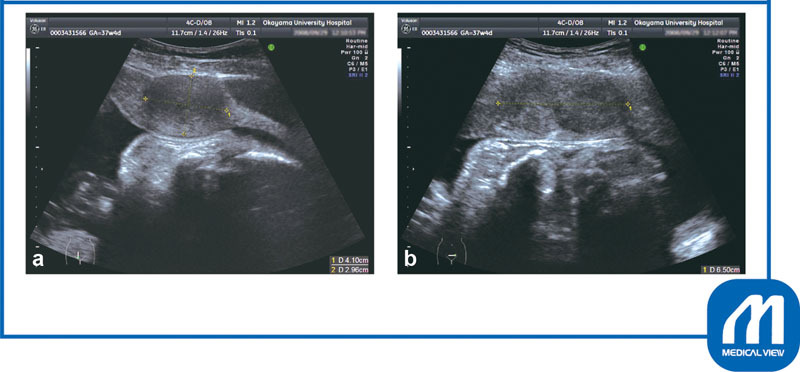
Case 2: ultrasonography findings. Two myomas (4-cm and 3-cm diameter) aligned in the anterior wall of the lower segment where the transverse incision is usually made. **(a)** Sagittal scan; **(b)** transverse scan. (Reproduced with permission of Hiramatsu Y. Cesarean section in patients with myomas located on the anterior wall of the cervix. In: Hiramatsu Y, Konishi I, Sakuragi N, Takeda S, eds. OGS Now, No.11 Uterine Myoma—How Do You Operate in Such a Case? (Japanese). Tokyo: Medical View; 2012:118–125. Copyright © Medical View).

This patient had myomas of 4-cm and 3-cm diameter aligned in the anterior wall of the lower segment where the transverse incision is usually made ( Fig. 8 ).

*Preoperative judgment* : In this patient, a transverse incision on the upper edge of the myoma or a transverse incision on the lower edge of the myoma was the option. However, choosing the first method, the incision would be made approximately 3 cm above the upper edge of the bladder and would extend to the uterine body. Therefore, we selected the following approach: (1) First, we planned a transverse incision slightly below the normal incision to try to deliver the fetus. (2) If this was difficult, we planned to add a reverse *T* -shaped incision between the two myomas.

**Fig. 9:**
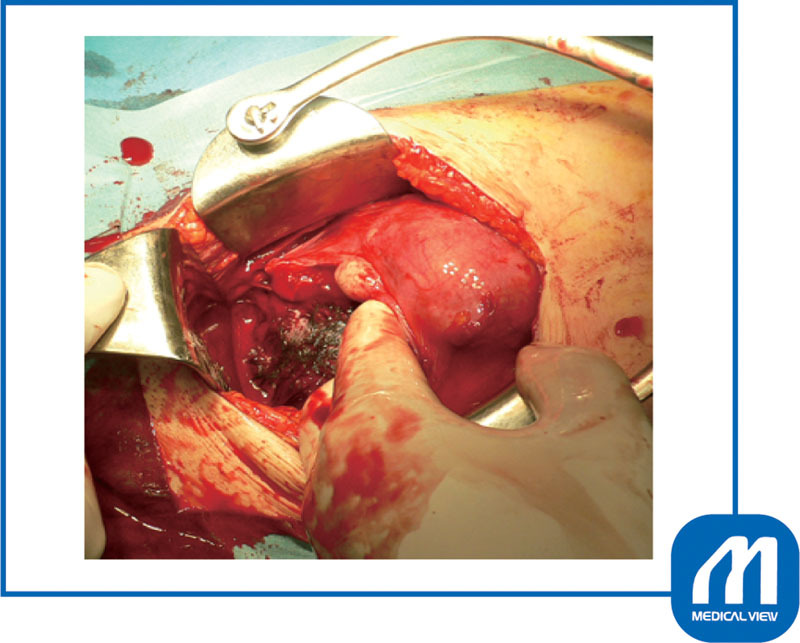
Case 2: condition after lower segment transverse incision. We made a transverse incision 1.5 cm below the usual cesarean section incision and attempted to deliver the fetus. However, this incision was not large enough, so we made a reverse *T* -shaped incision immediately between the two myomas ( *dot line* ) and delivered the fetus. (Reproduced with permission of Hiramatsu Y. Cesarean section in patients with myomas located on the anterior wall of the cervix. In: Hiramatsu Y, Konishi I, Sakuragi N, Takeda S, eds. OGS Now, No.11 Uterine Myoma—How Do You Operate in Such a Case? (Japanese). Tokyo: Medical View; 2012:118–125. Copyright © Medical View).

**Fig. 10:**
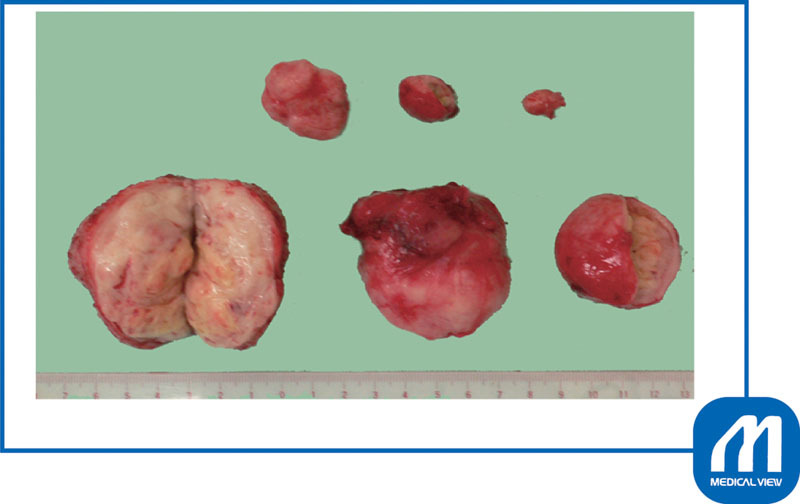
Case 2: enucleated myomas. Six myomas were enucleated. (Reproduced with permission of Hiramatsu Y. Cesarean section in patients with myomas located on the anterior wall of the cervix. In: Hiramatsu Y, Konishi I, Sakuragi N, Takeda S, eds. OGS Now, No.11 Uterine Myoma—How Do You Operate in Such a Case? (Japanese). Tokyo: Medical View; 2012:118–125. Copyright © Medical View).

*Surgical technique* : The bladder was dissected lower than usual, and we made a transverse incision 1.5 cm below the usual cesarean section incision and attempted to deliver the fetus. However, this incision was not large enough, and if we forced the fetal delivery, there was a risk of tearing the side wall and inducing major bleeding. At this point, we made a reverse *T* -shaped incision immediately between the two myomas ( Fig. 1d ), expanded the wound, and delivered the fetus ( Fig. 9 ). We then enucleated all six myomas ( Fig. 10 ).

### Important Point

In this case, the second procedure in our preoperative simulation was required. Once the fetal membranes rupture, amniotic fluid flows out, and the umbilical cord is compressed; therefore, speed is required. We must avoid situations where we determine the next intraoperative step only when we are unable to deliver a fetus using the first procedure.

### Case 3

**Fig. 11:**
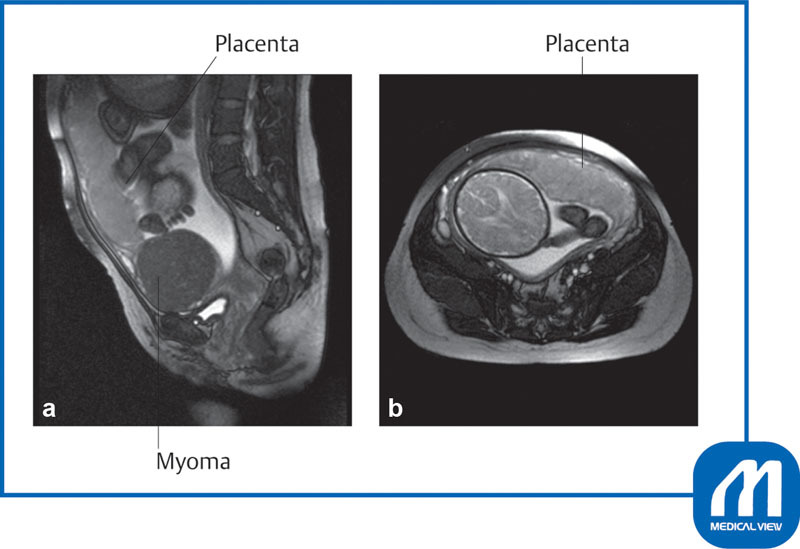
Case 3: myoma on anterior cervix magnetic resonance imaging findings (T2W). This patient had an 11-cm uterine myoma on the anterior wall of the cervix, which was in close contact with the placenta on the anterior uterine wall and extended to 11 cm above the umbilicus. **(a)** Sagittal scan; **(b)** horizontal scan. (Reproduced with permission of Hiramatsu Y. Cesarean section in patients with myomas located on the anterior wall of the cervix. In: Hiramatsu Y, Konishi I, Sakuragi N, Takeda S, eds. OGS Now, No.11 Uterine Myoma—How Do You Operate in Such a Case? (Japanese). Tokyo: Medical View; 2012:118–125. Copyright © Medical View).

**Fig. 12:**
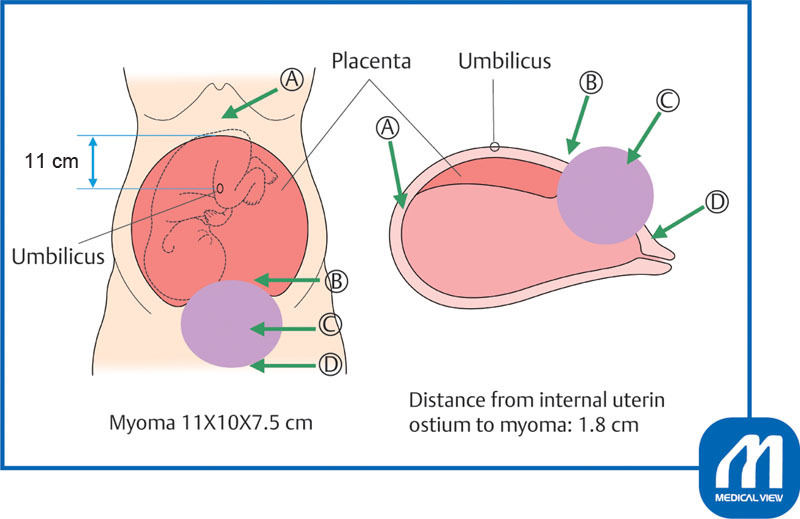
Schematic diagram of case 3. (Reproduced with permission of Hiramatsu Y. Cesarean section in patients with myomas located on the anterior wall of the cervix. In: Hiramatsu Y, Konishi I, Sakuragi N, Takeda S, eds. OGS Now, No.11 Uterine Myoma—How Do You Operate in Such a Case? (Japanese). Tokyo: Medical View; 2012:118–125. Copyright © Medical View).

This patient had an 11-cm uterine myoma on the anterior wall of the cervix, which was in close contact with the placenta on the anterior uterine wall and extended to 11 cm above the umbilicus ( Fig. 11 ). Fig. 12 shows a schematic diagram.

*Preoperative judgment* : In this case, we considered routes A to D as options to deliver the fetus ( Fig. 12 ). In most institutions, surgery is performed via route A, and many doctors chose route A in the preoperative conference for this patient. However, route A has the disadvantage of requiring a laparotomy opening to the xiphoid process. Route B might be suggested by a doctor experienced in delivering a fetus through the placenta in placenta previa. However, I felt this route should not be selected because if delivery is not possible using route B, it is not possible to immediately expand the wound downward. Route D is too low to secure enough space, and is considered likely to cause vascular or ureteral damage; therefore, I selected route C.

## Surgical Technique

I made a longitudinal incision from the pubic bone to 5 cm below the umbilicus, incised the serosa on the surface of the fibroma, and peeled the bladder downward. Next, I drilled a myoma borer into the myoma and pulled, enucleating the myoma. Finally, I made an incision in the remaining thin myometrium and delivered the fetus through the same wound as with a lower segment transverse cesarean section.

### Important Points

1. The uterine myoma was enucleated before delivering the fetus.
2. I enucleated the myoma without rupturing the fetal membranes.

## Pitfalls

If there is a myoma in the lower anterior uterine wall, the bladder may be elevated. It is necessary to check the upper edge of the bladder by ultrasonography and MRI with a full patient's bladder preoperatively. If the bladder is dissected without this preoperative knowledge, dissection can easily cause bladder damage.

## Points of Postoperative Management

- Treatment after fetal birth: Because uterine contractions are poor in pregnancy with myomas, the placenta is delivered while instilling a uterine contractor after delivering the fetus. In particular, if the placenta is attached to the myoma, intensive care is essential because contractions to expel the placenta are hindered and bleeding increases.
- If there is a myoma in the uterine isthmus or cervix, the cervical canal is often not dilated; therefore, it is necessary to dilate the cervix with a Hagar dilator.

## Notes

Surgical Steps

1. Laparotomy
  ↓
2. Uterine incision
  ↓
3. Deliver the fetus
  ↓
4. Placental delivery
  ↓
5. Suture the uterine incision
  ↓
6. Enucleate the myomas
  ↓
7. Irrigate the abdominal cavity
  ↓
8. Close the abdomen
